# Clinical Assessment of Weight Gain in Pediatric Patients Post-Tonsillectomy: A Retrospective Study

**DOI:** 10.7759/cureus.12005

**Published:** 2020-12-10

**Authors:** Zahrah A AlAbdullah, Khadijah Alali, Ibrahim Al Jabr

**Affiliations:** 1 Surgery, College of Medicine, King Faisal University, Al-Ahsa, SAU; 2 Miscellaneous, College of Medicine, King Faisal University, Al-Ahsa, SAU; 3 ENT, King Faisal University, Al-Ahsa, SAU

**Keywords:** weight gain, bmi, tonsillectomy, post-operative complications

## Abstract

Background

Tonsillectomy is one of the most commonly performed surgeries among children. It is indicated for the treatment of obstructive sleep apnea and chronic throat infections. Although a relatively safe surgery, post-operative complications have been reported in multiple studies. Over the past century, tonsillectomy has played a role in post-operative weight gain.

Aim

To measure weight gain in pediatric patients post-tonsillectomy.

Methods

A retrospective study was conducted in the Al-Ahsa region in Saudi Arabia. Overall, 240 children (male, 110 [44.2%]; female, 130 [52.2%]; mean±SD age, 7.45±2.89 years) from tertiary hospitals were included in the study. The height (m^2^) and weight (kg) of the children were measured, and the BMI (body mass index) was calculated preoperatively and one and six months post-operatively.

Results

There were significant differences observed between the mean weight one-month and six months post-operation (P = 0.0001) and the mean BMI measured at the baseline one month and six months post-operation (P = 0.0001). In addition, a positive linear correlation between the BMI six months post-operation and the weight at the same period (R = 0.375) was noted.

Conclusion

The findings of this study suggest an increase in weight post-tonsillectomy, mostly six months post-operation. Future studies, however, are warranted to evaluate the risk factors associated with weight gain in children and its relation to tonsillectomy.

## Introduction

Tonsillectomy is a commonly performed surgery in pediatric patients [1]. It has been practiced for many years and is considered the second most popular surgery performed in the United States among other surgeries [1]. When performed for reasonable indications, it improves patient quality of life and reduces the risk of airway obstruction [2-4]. Tonsillectomy is indicated in children with obstructive sleep apnea (OSA), usually performed with adjunctive uvulopalatoplasty. The relative indications include recurrent acute tonsillitis, peritonsillar abscess, and asymptomatic tonsillar hypertrophy. Although a relatively safe surgery, complications following the procedure have been globally documented [5]. Post-tonsillectomy hemorrhage is one of the most common and widely reported complications [6]. Others include taste impairment, respiratory compromise, dehydration, and secondary hemorrhage [7].

Children with recurrent acute tonsillitis suffer from reduced dietary intake. Studies have reported a rapid increase in weight gain post-tonsillectomy in some patients [8-11]. Recently, few retrospective studies reported a substantial increase in body mass index (BMI) post-tonsillectomy in children <6 years [12,13]. In some children, weight gain post-tonsillectomy may be attributed to energy reduction due to mouth breathing, decreased catecholamine, and increased oral intake [14-16]. The present article tries to bring light on the subject of the quality of life change after tonsillectomy. Mainly the previous articles focused on the indications of the surgical tonsillar removal, but not on the weight change.

## Materials and methods

A retrospective study was conducted in the Al-Ahsa region of Saudi Arabia between January 2018 and March 2019. Overall, 240 children aged 1 and 15 years from tertiary hospitals who underwent adenotonsillectomy were included in this study. Informed consent was obtained. Patient’s demographics include age, gender, residency, file number, and date and cause of the surgery. Height (m^2^) and weight (kg) of the children were measured preoperatively and one month and six months post-operatively. Furthermore, the BMI was calculated by dividing weight in kilograms over squared height (m^2^). The given values were compared to the growth chart of healthy Saudi children by percentiles based on age and gender: underweight (BMI <5th percentile), normal (BMI 5th-85th percentile), overweight (BMI 85th-95th percentile), and obese (BMI ≥95th percentile). The inclusion criteria were Saudi children aged 1 to 15 years who underwent adenotonsillectomy between January 2018 and March 2019 period for the following indications: OSA, recurrent acute tonsillitis, and tonsillar hypertrophy. Children with chronic disease and congenital malformation were excluded from the study. This study was approved by the research committee of King Faisal University, Al-Ahsa, Saudi Arabia.

Statistical analysis

The collected data were coded, tabulated, and statistically analyzed using IBM SPSS statistics, version 21 (IBM Corp., Armonk, NY). Descriptive statistics were used for quantitative data as the minimum and maximum range as well as the mean±SD for quantitative normally distributed data, while it was used for qualitative data as number and percentage. A correlation test was conducted between gender and BMI measurement. A P-value was 0.05 at a 90% confidence interval to determine the statistical significance.

## Results

Overall, 240 (male, 110 [44.2%]; female, 130 [52.2%]) children participated in this study to detect the change of mean weight pre- and post-tonsillectomy, according to the Saudi growth chart. The demographics of all included cases were as follows: age range, 1-15 years with a mean±SD of 7.45±2.89. The mean±SD of weight pre-operation, one month, and six months postoperation were 30.433±11.6, 30.53±12.05, and 33.25±12.27, respectively. Whereas the mean±SD of height pre-operation, one month, and six months post-operation was 1.14±0.18 m, 1.15±0.18 m, and 1.19±0.18 m. Regarding the BMI, the mean±SD of BMI pre-operation was 22.35±3.98, whereas the mean±SD of BMI one month and six months post-operation was 21.98±4.04 and 22.46±3.69, respectively. The characteristics of the participated children are shown in Table 1. 

**Table 1:** Demographics of the participated children

Variables	N(%)/ Mean±SD
Age (years) range	1-15
Age (years) Mean±SD	7.45±2.89
Male	110(44.2%)
Female	130(52.2%)
Weight pre-operation	30.433±11.6
Weight 1 month post-operation	30.53±12.05
Weight 6 months post-operation	33.25±12.27
Height pre-operation	1.14±0.18
Height 1 month post-operation	1.15±0.18
Height 6 months post-operation	1.19±0.18
BMI pre-operation	22.35±3.98
BMI 1 month post-operation	21.98±4.04
BMI 6 months post-operation	22.46±3.69

**Figure 1:**
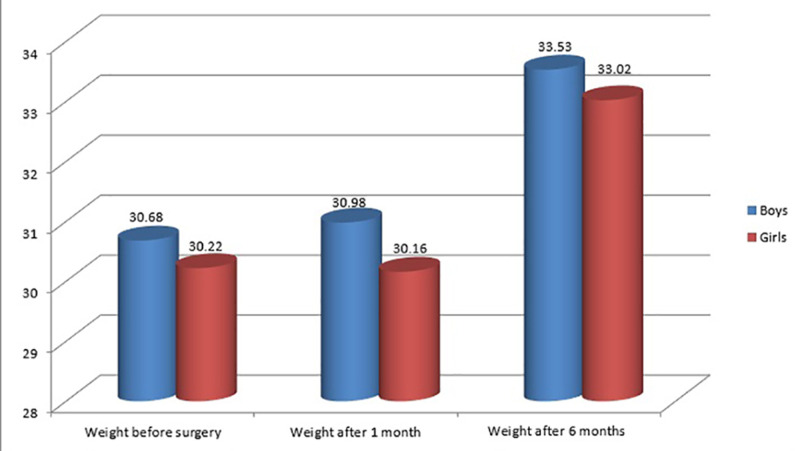
Weight of the participants

The mean weight pre- and post-operation for both genders are shown in Figure 1. In males, there was a slight increase observed in weight over the investigated durations, where the mean±SD of weight pre-operation, one month, and six months post-operation was 30.68±11.34, 30.98±11.68, and 33.53±11.94, respectively. Comparatively, in females, the mean±SD of weight pre-operation, one month, and six months post-operation was 30.22 ±11.93, 30.16±12.38, and 33.02±12.58, respectively

**Figure 2:**
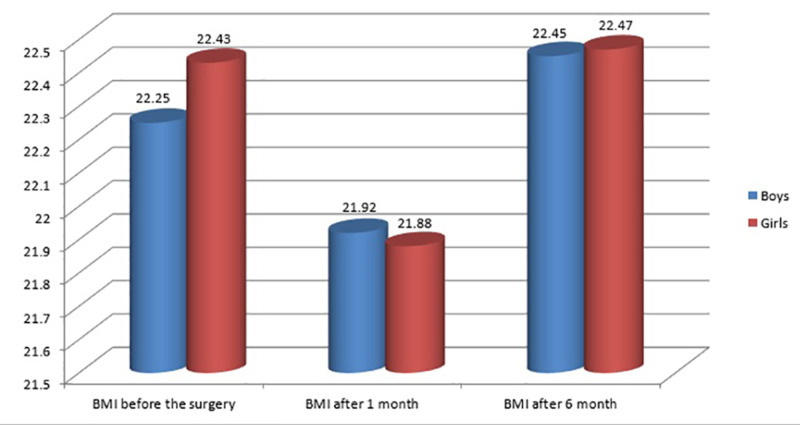
BMI of the participants

Figure 2 shows a comparison of the mean BMI between males and females. In males, the mean±SD of BMI was 22.25±3.7; there was a reduction in the BMI, one month post-operation 21.92±3.93, followed by an increase in the BMI six months post-operation 22.45±3.59. The same was found in females, where the mean±SD of BMI pre-operation, one month, and six months post-operation increased to 22.43±4.16, 21.88±4.5, and 22.47±3.79, respectively 

**Figure 3:**
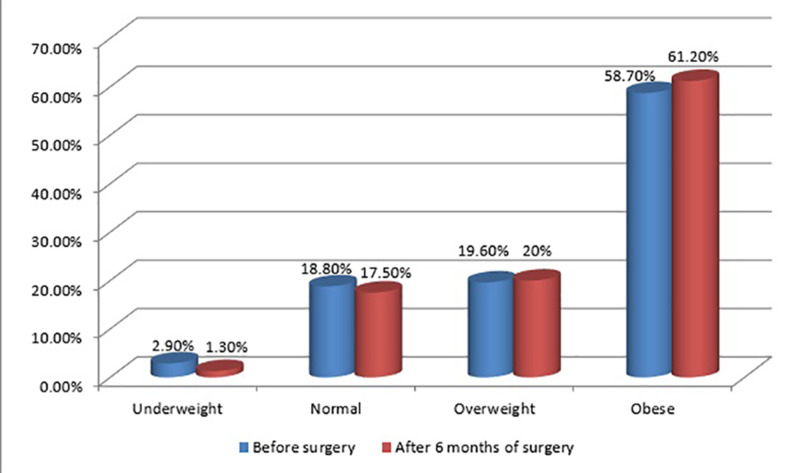
The classification of children according to the percentile growth chart pre- and six months post-operation

The classification of children according to the percentile growth chart pre- and six months post-tonsillectomy is shown in Figure 3. Preoperatively, there were 2.9%, 18.8%, 19.6%, and 58.7% underweight, normal, overweight, and obese children, respectively. However, six months post-operation, there were 1.3%, 17.5%, 20%, and 61.2% underweight, normal, overweight, and obese children, respectively.

There was a significant difference between the mean weight one month and six months post-operation (P < 0.0001). A repeated measure ANOVA test to investigate the differences in the weight at the baseline and one month and six months post-operation showed that there was a significant difference in the mean weight (P = 0.0001). There was a significant difference between the BMI measured at the baseline and one month and six months post-operation (P = 0.0001). In addition, there was a significant difference between the mean different percentile growth charts at the baseline and six months post-operation (P < 0.005). The Pearson correlation between the percentile growth chart and gender showed a significant correlation between these two variables (P = 0.034) pre- and post-operation (P < 0.013). Girls comprised the majority of the overweight group, whereas boys comprised the large majority of the obese group.

**Figure 4:**
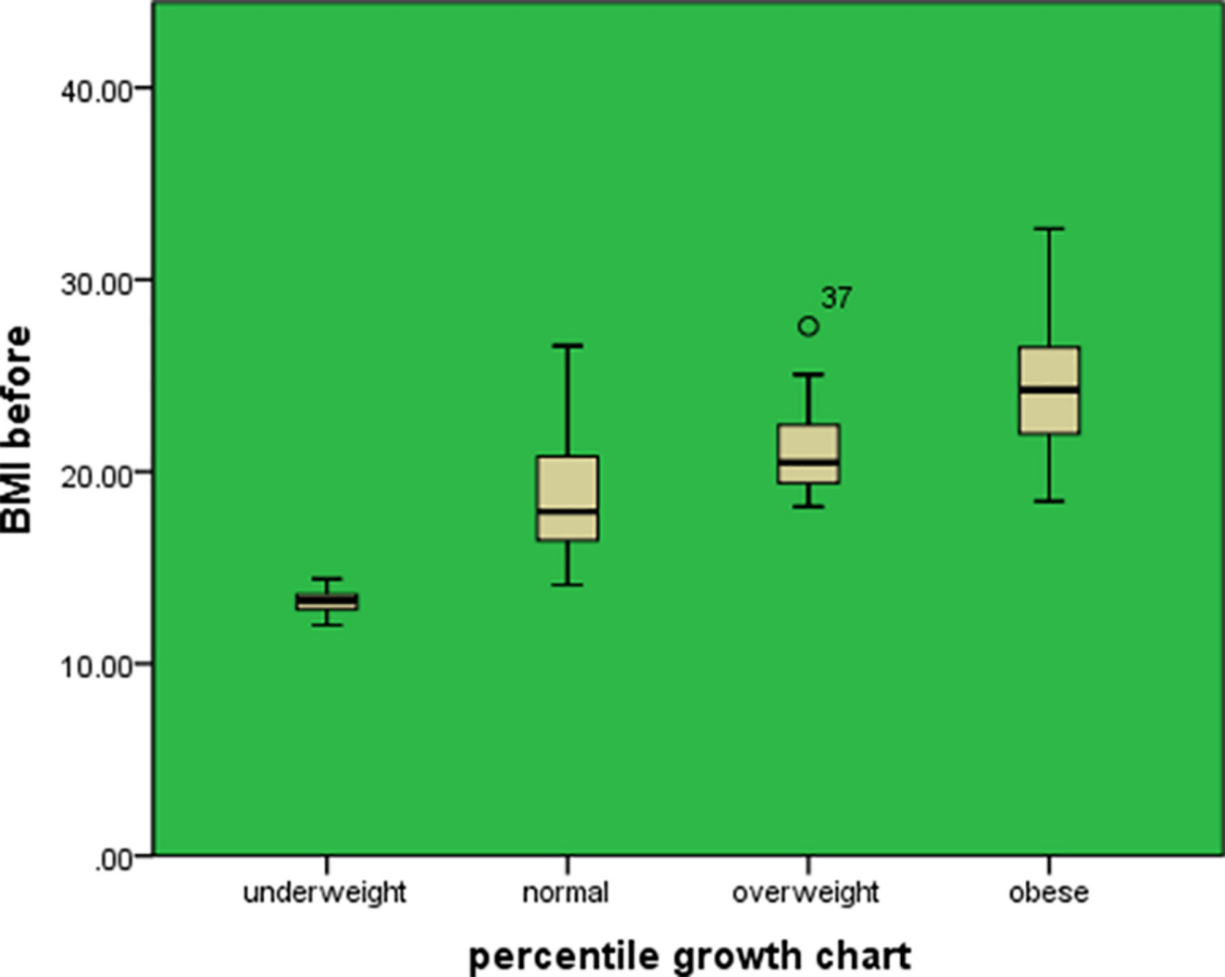
Box plot for percentile growth chart

**Figure 5:**
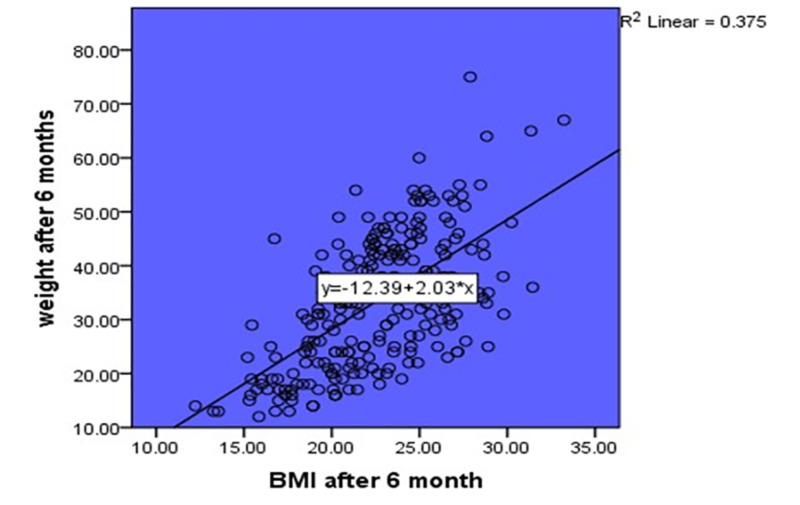
Scatter plot of the correlation between BMI and Weight showed a linear positive direct correlation

BMI at the baseline and after six months post-tonsillectomy can serve as a predictor for percentile growth chart before and after surgery with a statistically significant correlation (P = 0.000), with a 95% confidence interval (Figure 4). There was a positive linear correlation between BMI after six months of surgery and the weight at the same period (R = 0.375) (Figure 5).

## Discussion

In the present study, we collected and reviewed the data of 240 children who underwent a tonsillectomy at tertiary hospitals. The mean age of the children was 7.45±2.89 years old, with females more dominant than males. The mean weight of children after the surgery of one month increased and after six months increased in boys, whereas it decreased in girls. The mean of the overall weight increased after surgery compared to the weight before surgery; there was a significant difference between the weight at the baseline and after six months of the surgery. Also, there was a significant difference between the weight at baseline, one month, and six months after surgery. Regarding the height, the mean height in this study was increased after the surgery compared to the baseline before the surgery. 

A case-control study that included 154 children who underwent adenotonsillectomy [17 ] demonstrated that the surgery led to a significant increase in weight gain in obese children, but not in normal-weighted children. In this study, the mean of the overall BMI increased significantly post-operation compared to the baseline. Regarding the BMI classification of children, there was a significant difference in the distribution of children according to this classification at the baseline compared to six months post-operation. A significant correlation between gender and percentile growth chart pre- and post-operation was found, where girls tend to be overweight, whereas boys tend to be obese. Reportedly, the mean BMI of children increased significantly during follow-up compared to pre-operation [18], and this was in agreement with the findings of our study. A retrospective review that included children who underwent adenotonsillectomy found that many children gain more weight than expected post-operation. Moreover, the BMI increase was proportional to the BMI percentile pre-operation [19]. Contrary to our findings, a prospective controlled study that included children aged 2-14 years, comparable to the age group in our study, showed no significant difference in the mean of BMI between the cases and control groups during three months, six months, and one year post-operation [20]. Variation in the previous findings compared to our study can be attributed to several factors, such as diet variations of children in Saudi Arabia. Another study from Turkey showed that tonsillectomy in children had no effect on overweight and obesity, with no significant difference between the control group and the case group that underwent tonsillectomy. Another prospective study reported no significant difference between the control group and the case group regarding weight gain [20,21].

Another suggestion of the variation in the findings can be attributed to the study design. This is a retrospective study, and it only included children who underwent tonsillectomy, with no control group. We compared the increase in weight gain in the same group of children, whereas the other previous studies included a control group using a questionnaire [19-21], which was less accurate compared to using retrospective data of a hospital. This is because the information provided by the questionnaire can be affected by the participant’s opinion, whereas the data obtained from the hospital is based on clinical findings. The current study revealed that BMI could be a predictor for the percentile growth chart pre- and post-operation. A positive linear correlation between BMI and the weight 6 months post-operation was found.

## Conclusions

The findings of our study suggest that there is an increase in weight gain post-tonsillectomy. The difference is mostly observed in six months post-operation, with a positive linear correlation between weight and BMI. Further studies are warranted to evaluate the other risk factors associated with weight gain in children and its relation to tonsillectomy.

## References

[1] Baugh RF, Archer SM, Mitchell RB (2011). Clinical practice guideline: tonsillectomy in children. Otolaryngol Head Neck Surg.

[2] Sargi Z, Younis RT (2007). Tonsillectomy and adenoidectomy techniques: past, present and future. ORL.

[3] Lemkens N, Lemkens P, Egondi TW (2010). Antibiotic use and doctor visits are reduced after adenotonsillectomy. B-ENT.

[4] Piessens P, Hens G, Lemkens N, Schrooten W, Debruyne F, Lemkens P (2012). Effect of adenotonsillectomy on the use of respiratory medication. Int J Pediatr Otorhinolaryngol.

[5] Goyal SS, Shah R, Roberson DW, Schwartz ML (2013). Variation in post‐adenotonsillectomy admission practices in 24 pediatric hospitals. Laryngoscope.

[6] Belyea J, Chang Y, Rigby MH, Corsten G, Hong P (2014). Post-tonsillectomy complications in children less than three years of age: a case-control study. Int J Pediatr Otorhinolaryngol.

[7] Bonuck K, Parikh S, Bassila M (2006). Growth failure and sleep disordered breathing: a review of the literature. Int J Pediatr Otorhinolaryngol.

[8] Kaiser AD (1922). Effect of tonsillectomy on general health in five thousand children. JAMA.

[9] Paterson D, Bray GW (1928). Tonsillar hypertrophy and infection as a factor in iII-health. Lancet.

[10] Hodges S, Wailoo MP (1987). Tonsillar enlargement and failure to thrive. Br Med J (Clin Res Ed).

[11] Amin R, Anthony L, Somers V (2008). Growth velocity predicts recurrence of sleep-disordered breathing 1 year after adenotonsillectomy. Am J Respir Crit Care Med.

[12] Smith DF, Vikani AR, Benke JR, Boss EF, Ishman SL (2013). Weight gain after adenotonsillectomy is more common in young children. Otolaryngol Head Neck Surg.

[13] Levi J, Leoniak S, Schmidt R (2012). Evaluating tonsillectomy as a risk factor for childhood obesity. Arch Otolaryngol Head Neck Surg.

[14] Gkouskou KK, Vlastos IM, Hajiioannou I, Hatzaki I, Houlakis M, Fragkiadakis GA (2010). Dietary habits of preschool aged children with tonsillar hypertrophy, pre-and post-operatively. Eur Rev Med Pharmacol Sci.

[15] Nieminen P, Löppönen T, Tolonen U, Lanning P, Knip M, Löppönen H (2002). Growth and biochemical markers of growth in children with snoring and obstructive sleep apnea. Pediatrics.

[16] Roemmich JN, Barkley JE, D'Andrea L, Nikova M, Rogol AD, Carskadon MA, Suratt PM (2006). Increases in overweight after adenotonsillectomy in overweight children with obstructive sleep-disordered breathing are associated with decreases in motor activity and hyperactivity. Pediatrics.

[17] Lewis TL, Johnson RF, Choi J, Mitchell RB (2015). Weight gin after adenotonsillectomy: a case-control study. Otolaryngol Head Neck Surg.

[18] Czechowicz JA, Chang KW (2014). Analysis of growth curves in children after adenotonsillectomy. JAMA Otolaryngol Head Neck Surg.

[19] Al Abdulla AF, Prabhu S, Behzad KE (2018). Prospective controlled study on post-tonsillectomy weight gain-by objective and subjective methods. Egypt J Ear Nose Throat Allied Sci.

[20] Topal K, Kara CO, Bozkurt AI, Saatci E (2013). The risk of overweight and obesity in children after tonsillectomy: a cross-sectional study. Eur Arch Otorhinolaryngol.

[21] Al Kindy SA, Alzahrani AK (2018). Tonsillectomy and weight gain in children: a prospective study. Saudi Surg J.

